# Primary Central Nervous System Lymphoma in Elderly Patients: Management and Perspectives

**DOI:** 10.3390/cancers13143479

**Published:** 2021-07-12

**Authors:** Andrea Morales-Martinez, Fernando Lozano-Sanchez, Alberto Duran-Peña, Khe Hoang-Xuan, Caroline Houillier

**Affiliations:** 1 APHP, Groupe Hospitalier Salpêtrière, Sorbonne Université, IHU, ICM, Service de Neurologie 2-Mazarin, 75013 Paris, France; dra.a.morales@gmail.com (A.M.-M.); fer_alsmd86@hotmail.com (F.L.-S.); betitodu@gmail.com (A.D.-P.); khe.hoang-xuan@aphp.fr (K.H.-X.); 2 LOC Network, 75013 Paris, France

**Keywords:** primary CNS lymphoma, elderly, chemotherapy, immunotherapy

## Abstract

**Simple Summary:**

We present a review of the most relevant epidemiological, diagnostic and therapeutic aspects of primary central nervous system lymphoma (PCNSL) in elderly patients.

**Abstract:**

The management of elderly patients suffering from primary central nervous system (CNS) lymphoma, who represent a rapidly growing population, is challenging. Despite the advances made in PCNSL treatment, the prognosis in older patients remains unsatisfactory. The high risk of systemic and CNS toxicity induced by a high-dose chemotherapy regimen and radiation therapy, respectively, limits the use of consolidation phase treatments in elderly patients and contributes to the poor outcome of these patients. Here, we review the current treatment strategies and ongoing trials proposed for elderly PCNSL patients.

## 1. Introduction

Primary central nervous system lymphoma (PCNSL) is a highly aggressive non-Hodgkin lymphoma confined to the central nervous system (CNS) and mainly affects elderly patients. Even when there is a not clear age cutoff, the ages of 60–65 years old were widely considered the cutoff range for defining the elderly population in most studies assessing the correlation of worse outcomes and increased risk of treatment-related neurotoxicity. National series in Europe and the United States report a median age of approximately 67 years, while the prevalence of patients older than 60 years in these series is reported to be between 60 and 70% [1,2,3,4,5,6].

Studies carried out with different nationwide population cohorts have revealed an increased incidence among elderly patients over time, both overall and in the immunocompetent subgroup [1,2,3,4,5]. Large population studies in the United States have shown an increase in the PCNSL rate of 1.7% per year in people older than 65 years (with stable rates in other age groups) during the period 1992–2011 [1] and an increase in the incidence, from 0.2 in 1973 to 2.1 per 100,000 in 2013, in people older than 70 years [3]. In addition, a population-based study in Sweden revealed an increased incidence, from 0.43 to 1.66 per 100,000 habitants among patients aged 70 and older between 2000–2002 and 2012–2013 [2]. A nationwide population-based study reported an overall increase among PCNSL patients diagnosed in the Netherlands between 1989 and 2015 as a result of the increasing incidence in the group older than 60 years, exhibiting a twofold increase in PCNSL incidence during this period [4]. This increase remains largely unexplained even though it may be partially due to the global aging of the population, longer life expectancy and advances in diagnostic techniques and approaches.

PCNSL overall survival (OS) has steadily improved for the youngest patients. In contrast, despite advances in the management of the older population, the prognosis of elderly patients remains poor [2,3,4,7]. An American nationwide report showed that PCNSL survival has not improved for patients older than 70 years since the 1970s, remaining at approximately 6–7 months, even though the median OS of all patients doubled in the same period [3]. However, the results from nationwide reports include patients treated only with palliative care, a common situation for elderly individuals, which is not always reported. A Dutch nationwide population-based study revealed that, while the use of chemotherapy has progressively increased in elderly patients up to age 70 years, with a consequent improvement in OS, approximately 40% of patients older than 70 years did not receive antineoplastic therapy, exhibiting poor prognosis [4]. A systematic review and meta-analysis by Kasenda et al. of 783 elderly patients diagnosed with and treated for PCNSL from 1977 to 2014 revealed a progressive OS improvement over time [7].

In different series and clinical trials, where the objective response rate (ORR) to treatment (chemotherapy and/or radiotherapy) according to age group was available, the elderly group exhibited a lower complete response (CR) rate, which was reported to be approximately 30–60%, versus 50–70% for younger patients [6,8,9,10,11,12,13,14]. Similarly, reports showed a progression-free survival (PFS) after CR that was much lower in the older group, between 8 and 16 months, versus 28 to 35 for the younger group [6,8]. Notably, as reported in a large prospective nationwide cohort of more than 1000 consecutive newly diagnosed PCNSL patients, elderly patients were particularly exposed to the risk of early death (i.e., during the first six months after diagnosis), affecting 25% of patients [6]. Interestingly, more than one-half of these early deaths were not related to resistance to chemotherapy but to comorbid complications and treatment-related toxicity, exacerbated by precarious health and poor neurological condition. This finding indicates the importance of better individualized treatment for this fragile population.

Regarding 1st line treatment, elderly patients are more often untreated or less vigorously treated than younger patients [4,6,8,14,15]. Altogether, physiological status related to age, higher rate of comorbidities and differences in therapeutic management may contribute to the poorer prognosis in elderly patients with PCNSL and warrant to be better investigated and taken into account, in order to optimize management strategies and tolerance and improve outcomes.

This evidence supports the development of clinical trials dedicated to elderly patients with PCNSL, considering the specific risks of this age group. This review focuses on the main management issues and therapeutic perspectives for elderly patients with PCNSL.

## 2. Clinical Aspects

Functional status at the time of diagnosis, an independent prognostic factor in PCNSL [16,17,18], is usually lower in elderly patients than it is in younger patients [6,14]. Clinical manifestations in elderly patients are similar to those in younger patients, including focal neurological deficits, neurocognitive and/or behavior changes, symptoms of increased intracranial pressure and, less frequently, epilepsy. However, elderly patients display a higher proportion of cognitive impairments at diagnosis than those in other age groups [6,14,19]. Unpublished data from the French LOC network study show a higher proportion of cognitive impairments at the time of diagnosis in patients older than 60 years (65% versus 48%, *p* < 0.001). In elderly patients, cognitive symptoms could be inaccurately attributed or associated with other prevalent pathologies in this age group (such as vascular or degenerative diseases), increasing the delay in diagnosis [19] and affecting the prognosis [14,20,21]. In addition, neurocognitive dysfunction at diagnosis was recently reported as an independent prognostic factor [22,23].

Older age is also associated with a higher frequency of comorbidities, which may increase the risk of therapy-induced toxicity that alters the pharmacokinetics and pharmacodynamics of therapy and may increase the risk of toxicities. However, as discussed below, elderly patients with PCNSL can achieve a response even to more intense treatments, so they should not be excluded from these therapies only on the basis of age criteria, but rather on the basis of a global assessment for fitness. Nevertheless, this approach is still very scarce. In a pilot study, Schorb et al. have utilized the Cumulative Illness Rating Scale–Geriatric score as an inclusion criterion in addition to age [24]. Such approaches should be developed.

## 3. Diagnosis

The PCNSL diagnostic approach in the elderly population is similar to that of other age groups. Histopathological confirmation by cerebral stereotactic biopsy or positive cytology in the CSF or vitreous biopsy sample is required to establish the diagnosis before starting treatment and should be obtained without delay [25,26]. Because steroids may induce rapid tumor shrinkage [27,28,29,30,31,32], their use before brain biopsy should be delayed as much as possible as it may prevent pathological confirmation [25,26].

Stereotactic biopsy is considered a safe high-yield diagnostic procedure in PCNSL [33], even in elderly patients and in cases of deep lesions that are quite common [34,35,36]. However, elderly patients present more frequently with deteriorated functional status and/or the presence of multiple comorbidities, preventing the use of biopsy to obtain samples. In these situations, a noninvasive diagnostic tool with high sensitivity and specificity would be useful.

In the past decade, several biomarkers measured in the CSF and the vitreous (in the case of vitreoretinal involvement) were investigated as potentially useful for the diagnosis of PCNSL, such as IL-10, IL-6, CXCL13, miRNA 19-21-92a, neopterin, CD19, and MYD88 hotspot mutations [37,38,39,40,41,42,43,44,45,46,47,48,49,50]. Although biopsy remains the gold standard for diagnosis, the increase in the CSF IL-10 level and CSF IL-10/IL-6 ratio, with sensitivities ranging from 60 to 97% and specificities ranging from 90 to 100% [43,44,45,46,47], are helpful tools for diagnostic guidance, especially in atypical radiological presentations and when performing a biopsy is not feasible. Preliminary results also suggest their potential predictive role in posttreatment evaluation for monitoring treatment responses [45]. A combined analysis of MYD88 mutation and IL-10 level in the CSF was reported with a sensitivity and specificity of 94% and 98%, respectively, in newly diagnosed PCNSL [50].

## 4. Treatment

Even though PCSNL is known to be highly sensitive to therapy, its optimal management remains controversial because of the difficulty of carrying out large, randomized studies in an age population subgroup of a low-incidence disease. Treatment of PCNSL consists of a two-phase treatment: an induction phase based on polychemotherapy to achieve a CR, followed by a consolidation phase when possible, to prevent relapse and to improve outcome with a curative objective.

### 4.1. Achieving a High Rate of Remission: Induction Treatment

A polychemotherapy protocol based on HD-MTX as the principal agent, combined with other drugs known to cross the blood–brain barrier, is widely considered the optimal induction treatment in younger and elderly patients with adequate cardiac and renal functions [25]. HD-MTX is defined as a minimal dose of 0.5 g/m^2^ [51] to cross the blood–brain barrier; however, the recommended dose is at least 3 g/m^2^ delivered in 3 h in order to yield cytotoxic levels in the CSF [25,26,52].

Most studies devoted to HD-MTX-based chemotherapy alone as a 1st line treatment in elderly patients with PCNSL are retrospective studies, with only a few single-arm phase II studies, a single randomized phase II study and no phase III studies. Hence, many questions with respect to this population remain unanswered, such as the optimal MTX dose and dose interval, number of MTX infusions, and the best drugs to be used in combination with HD-MTX. The main studies are summarized in Table 1. Various other cytotoxic agents were associated with HD-MTX, notably procarbazine, vincristine, lomustine, high-dose cytarabine or temozolomide. The CR rate ranged between 17 and 69%, with a median PFS between 6 and 16 months and a median OS between 14 and 37 months.

In the meta-analysis by Kasenda et al., based on 22 studies published between 1996 and 2014 including 783 elderly patients with a median age of 68 years, 1st line HD-MTX-based chemotherapy was used in 573 patients [7]. After a median follow-up of 40 months, the authors found that HD-MTX-based therapy was associated with an improved ORR (73% versus 55%; OR 1.60, 95% CI: 0.98–2.62) and OS (HR 0.70, *p* = 0.013) compared with therapies without HD-MTX, including radiotherapy alone. HD-MTX combined with any other chemotherapy had a significantly higher ORR than HD-MTX alone (73% versus 68%; OR 3.63, 95% CI: 1.51–8.68) and improved PFS (HR 0.39, *p* < 0.001). HD-MTX combined with oral chemotherapy (e.g., HD-MTX combined with temozolomide or procarbazine) compared to other more aggressive protocols (HD-MTX plus at least two other intravenous agents) had similar ORRs and OS rates.

In the randomized phase II study conducted by the ANOCEF-GOELAMS, 98 patients aged over 60 years were randomized to receive either an association of HD-MTX, procarbazine, vincristine and cytarabine (MPVA), or an association of HD-MTX and temozolomide (MT) [22]. Although this trial was not designed for direct comparison between both arms, a trend to better outcomes was found with MPVA: ORR of 82% versus 71%, median PFS of 9.5 versus 6.1 months and median OS of 31 versus 14 months, respectively. No significant differences were noted in toxicity between the two groups. Of interest, both arms were associated with an improvement and/or preservation over time of quality of life and neurocognitive functions evaluated by neuropsychological testing, supporting the value of this approach in the elderly.

HD-MTX exposes patients to a risk of potentially life-threatening complications, including acute renal insufficiency, toxic hepatitis, myelosuppression and infections. However, in the ANOCEF-GOELAMS trial, toxicities were acceptable and manageable in most cases. The most frequent were liver dysfunction and lymphopenia, and both were reversible. Grade III/IV renal toxicity was less frequent, occurring in less than 10% of patients. Dose reduction of MTX attributable to toxicity was reported in approximately 30% of patients. Toxic deaths were reported in 8% of patients, mostly because of sepsis [22]. Similar results were reported in other prospective studies dedicated to the elderly [9,55,56,63].

Hence, automatically reducing the MTX dose simply according to the patient’s age is not justified, especially in light of the poor prognosis of elderly patients. Special attention should be given to the prevention of MTX accumulation and subsequent potential toxicities, especially alkaline hyperhydration prior to MTX infusion and folinic acid rescue following infusion. In addition, HD-MTX must be monitored with dose reduction schedules based on the urinary clearance rate before each treatment cycle [63]. As a last resort, glucarpidase (carboxypeptidase-G2) can be used to reduce very rapidly an important increase in the serum concentration of methotrexate [64].

Rituximab, a standard treatment in systemic B-cell lymphoma, remains controversial as a treatment for PCNSL [65]. A randomized phase II trial with patients aged between 18 and 70 years, with no specific data for elderly patients, showed a significantly higher CR rate when rituximab was added to HD-MTX and cytarabine combination therapy [66]. In elderly patients, retrospective studies have shown encouraging results with HD-MTX-based regimens associated with rituximab compared to the same regimens without rituximab [11,55,56]. However, in a recent phase III study including patients aged 18 to 70 years old (median age: 61 years old), there was no benefit in terms of PFS or OS when rituximab was added to an HD-MTX-based polychemotherapy regimen (MBVP: MTX, BCNU, and VM26). Of note, consolidation strategy changed according to age: after induction, all responding patients received consolidation chemotherapy with cytarabine and those aged 60 years or younger received additional WBRT; patients older than 60 years did not receive radiotherapy [67]. Moreover, in a post hoc subgroup analyzed by age, the patients older than 60 years treated in the arm with rituximab tended to have a poorer outcome (nonsignificant): median PFS was 19.5 versus 14.6 months and median OS was 49.2 versus 34.9 months, respectively. However, these results have to be considered with caution, as they represent results from an unplanned analysis.

#### Intrathecal Chemotherapy

HD-MTX penetrates the meninges well and the use of intrathecal treatment (MTX and cytarabine) in patients with lymphomatous meningitis treated with HD-MTX has not been established in the absence of controlled trials. If used, these drugs are likely delivered through an intraventricular Ommaya reservoir with a reservoir infection rate of 9% [68]. This approach was proposed in some chemotherapy regimens, including those used with elderly patients [9,68,69,70], and may be discussed when intravenous MTX is administered at doses below 3 g/m^2^, to allow reaching cytotoxic levels in the CSF.

### 4.2. Achieving Long-Term Remission

#### 4.2.1. Consolidation Treatment

The objective of consolidation treatment is to prevent the risk of relapse in patients who have achieved remission after an induction phase and to improve outcomes. However, in the elderly population, the options for a consolidation phase are very limited because of the risk of toxicity in this frail population.

##### Whole-Brain Radiation Therapy (WBRT)

WBRT used alone as a 1st line treatment has been disappointing, providing limited survival benefit in PCNSL patients, especially in older patients. In a phase II trial evaluating WBRT at a total dose of 40 Gy with a booster of 20 Gy, the cases of remission were of short duration, and the median OS was only 7.2 months in patients older than 60 years [71]. Historically, combination treatment consisting of HD-MTX-based induction chemotherapy followed by WBRT consolidation was considered by broad consensus as the standard of care in the 1990s. However, the combination of HD-MTX and WBRT at the classic dose (total dose: 40–45 Gy; dose per fraction: 1.8–2 Gy) is associated with a risk of central neurotoxicity leading to delayed progressive cognitive dysfunction, ataxia and urinary incontinence, with a typical aspect of leukoencephalopathy and brain atrophy on MRI scans. This risk is particularly important in patients older than 60 years, and it was reported to be as high as 80% in some studies using combined treatment [72,73]. This adverse event occurs earlier [74] and more severely in older than in younger patients, with patients eventually becoming bedridden or developing severe dementia [72,74,75,76,77].

The results of the G-PCNSL-SG 1 phase III trial suggested that the omission of WBRT as a consolidation treatment after HD-MTX as 1st line therapy does not compromise OS. A post hoc analysis found the same result for complete responder patients aged 70 or older, with an OS of 29.3 months with WBRT versus 26.7 without WBRT [8]. In his meta-analysis devoted to older patients, Kasenda et al. found no differences in remission rates between patients treated with HD-MTX-based chemotherapy with or without WBRT as 1st line therapy, while WBRT was an independent risk factor for neurological side effects (adjusted OR 5.23; *p* < 0.001) [7]. The high risk of severe neurotoxicity appeared unacceptable in patients older than 60 years, causing consolidation WBRT at classic doses to be progressively abandoned in the 2000s.

The use of lower doses is a potential alternative approach to WBRT omission to reduce delayed treatment neurotoxicity. In a phase II trial enrolling 52 newly diagnosed PCNSL patients (median age 60 years, range from 30 to 79 years) and evaluating an immunochemoradiation regimen, including rituximab and HD-MTX-based polychemotherapy (MPVA), 31 complete responders to induction were treated with reduced-dose WBRT (23.4 Gy in 13 fractions) with encouraging results, both in terms of survival and neurotoxicity [78]. In the older subgroup, the outcome was promising, with a median PFS of 4.4 years, while the median OS was not reached with a median follow-up of 5.9 years. However, formal neuropsychological testing to evaluate the delayed neurotoxicity reliably in this age population subgroup was applied to only three elderly CR patients. These results prompted the authors to set up a randomized phase II clinical trial comparing the RMPVA chemotherapy regimen with or without low-dose WBRT with special attention to treatment-related neurotoxicity rates and disease-related cognitive deterioration in each arm through prospective neuropsychological evaluation (ClinicalTrials.gov Identifier: NCT01399372, Table 2).

##### High-Dose Chemotherapy Conditioning with Autologous Stem Cell Transplantation (HDC-ASCT)

HDC-ASCT following an induction chemotherapy strategy has shown high rates of long-term remission in younger patients with PCNSL, in relapsing/refractory patients [79] and more recently in 1st line treatment [13,80,81,82]. In randomized trials, this strategy has been shown to be an alternative option to WBRT as consolidation, with a better preservation of neurocognitive functions at least in the short-term follow-up, but at the price of an expected important systemic toxicity and a toxic death rate of 5–10%. While this strategy has been classically reserved for younger patients, a few recent studies have supported its use in selected older patients with age-adapted protocols [24,59,82].

A retrospective multicenter study investigated the outcomes of thiotepa-based HDC-ASCT in 52 elderly patients (median age 68.5 years, range from 65 to 77 years), with a median Karnofsky Performance Status (KPS) before HDC-ASCT of 80% (range 30–100%), who were treated in 1st (*n* = 15) or 2nd or subsequent line (*n* = 37) [59]. The ORR was 86.5% (93% in 1st line and 83.6% in 2nd or subsequent line). The 2-year PFS and OS rates were 62% (80% in 1st line, 54% in 2nd or subsequent lines) and 70.8% (80% in 1st line, 65.6% in 2nd or subsequent lines), respectively. The rate of toxic-related death was 3.8% (*n* = 2). In a single-arm prospective pilot trial, 13 patients older than 65 years (range from 70 to 79 years) who had achieved CR or partial response (PR) after induction with rituximab, HD-MTX and cytarabine received a consolidation protocol of HD-ASCT based on busulfan IV at 3.2 mg/kg and thiotepa IV at 5 mg/kg [24]. The inclusion criteria included the following: Eastern Cooperative Group Performance Status (ECOG-PS) ≤ 2, Cumulative Illness Rating Scale–Geriatric score < 6 (excluding direct PCNSL symptoms), and adequate bone marrow, hepatic and renal function (creatinine clearance ≥ 60 mL/min). The results of this regimen in selected elderly patients were excellent, with 2-year PFS and OS rates of 93% and 92.3%, respectively. After a median follow-up of 41 months, 12 of 13 patients retained their CR with good mental status and general condition. An ongoing German multicenter phase II clinical trial seeks to confirm these results, evaluating the feasibility and efficacy of an age-adapted induction treatment followed by HDC-ASCT in fit patients aged older than 65 years (DRKS00011932, Table 2).

If short-term follow-up suggests that HDC-ASCT and low-dose WBRT approaches are associated with reduced neurocognitive decline compared to standard total-dose WBRT, prudence should prevail and further studies regarding the long-term neurocognitive outcomes should be completed and the results considered. A longitudinal cognitive assessment was conducted on 29 patients with newly diagnosed PCNSL treated by consolidation agents with reduced-dose WBRT (median age 58 years, range from 49 to 76 years) or HDC-ASCT (median age 53 years, range from 24 to 68 years) as part of two prospective phase II clinical trials at Memorial Sloan Kettering Cancer Center (MSKCC), with no evidence of disease progression 5 years posttreatment [83]. Longitudinal follow-up of completed serial cognitive evaluations showed a continuous improvement from baseline up to year 3, followed by a decline in cognitive functions in both groups without significant longitudinal differences between the two groups on any of the cognitive tests. The incidence of white matter (WM) disease and cortical atrophy (CA) increased over time in both groups. Even when these brain structure abnormalities were more frequent in patients treated with rdWBRT, there were no significant longitudinal differences between the two groups in WM or CA ratings. These results suggest that both rdWBRT and HDC-ASCT may be associated with delayed neurotoxicity in progression-free patients. Hence, prospective neuropsychological testing will be important to perform in future HDC-ASCT trials dedicated to elderly individuals.

##### Nonmyeloablative Intensive Chemotherapy

A retrospective study devoted to elderly patients failed to show a clear benefit of extending cytarabine consolidation treatment after methotrexate-based chemotherapy (R-MPV regimen consolidated with three cycles of high-dose cytarabine instead of one) but was associated with increased toxicity in elderly patients [11].

The CALGB50202 phase II trial reported better results of a consolidation regimen combining high-dose etoposide and cytarabine (EA) in patients up to 76 years old after rituximab, methotrexate and temozolomide (RMT) induction [84]. These encouraging results were confirmed in an independent single-center series of 28 consecutive newly diagnosed patients with PCNSL, including a majority of older patients. However, EA was much more toxic than previously reported, especially regarding the length of neutropenia and related infectious complications [85].

A recent preliminary report of a randomized phase II trial, showed non-significant differences in PFS landmarked at start of consolidation by HDT/ASCT with thiotepa and carmustine or non-myeloablative chemotherapy with cytarabine and etoposide, in 108 patients with a median age of 61 years (range 33–75), with similar toxicities between arms and no treatment-related mortality during consolidation [86].

#### 4.2.2. Maintenance Treatment

Maintenance treatment may be a relevant alternative approach for elderly patients, especially for those who are not fit for vigorous consolidation therapy (WBRT or intensive chemotherapy with or without ASCT). Different maintenance chemotherapy regimens were evaluated in retrospective or single-arm prospective trials using HD-MTX, temozolomide, CCNU, procarbazine, and rituximab as single agents or in combination. Table 1 summarizes the studies on maintenance treatment (following any induction schema) including elderly patients. The CR after a maintenance therapy varied between 55 and 85%, achieving a conversion from PR to CR between 70 and 100%. These preliminary studies show that the use of maintenance therapy is a feasible option in this age group. A randomized phase III clinical trial conducted by the French LOC network evaluating the role of maintenance therapy in patients aged 60 years or older is currently ongoing (BLOCAGE trial, NCT02313389, Table 2). After an induction phase including rituximab plus the MPVA chemotherapy regimen in patients aged 60 years or older, complete responders are randomized to receive either maintenance therapy (RMT regimen: rituximab, HD-MT, and temozolomide) or observation. Cognitive function, quality of life, and oncogeriatric scales are evaluated by a standardized and validated battery of tests and questionnaires as part of the secondary outcome measures.

Interestingly, novel agents that have shown activity in PCNSL with a good safety profile from the perspective of prolonged treatment may be used in maintenance treatment as single agents or in combination with other drugs (see Table 1). Hence, a retrospective analysis of 13 elderly patients treated with lenalidomide as maintenance treatment after induction chemotherapy with HD-MTX, temozolomide and rituximab [62], led to persistent survival outcomes, with a median PFS not reached after a median follow-up exceeding 30 months. Lenalidomide in association with rituximab is currently being evaluated in a phase II trial as maintenance therapy (NCT04627753, Table 2). In another phase II trial, fit patients will be randomized after standard induction polychemotherapy, to receive either procarbazine or lenalidomide as maintenance therapy, while unfit patients will receive WBRT plus temozolomide and rituximab as induction therapy, followed by temozolomide as maintenance treatment (NCT03495960: FIORELLA, Table 2). A phase II study using ibrutinib as a single agent for maintenance was also established (NCT02623010, Table 2).

### 4.3. Salvage Treatment

Two small retrospective series, with approximately one-half of the patients older than 60 years [87,88], reported encouraging results with HD-MTX-based chemotherapy rechallenge in patients who had previously achieved CR with HD-MTX-based therapy (the ORR was 85 and 91%, median PFS was 16 months and 25.8 months and median OS of 41 months and 62 months). Hence, HD-MTX rechallenge may be effective in patients who have retained chemosensitivity to MTX despite previous exposure to the drug. Based on retrospective studies, other salvage therapies using conventional agents include a combination of procarbazine, lomustine and vincristine [89], temozolomide alone or with rituximab [90,91,92], pemetrexed [93,94,95,96], topotecan [97,98], platinum-based polychemotherapy [99], a combination of etoposide, ifosfamide and cytarabine [100], and a combination of ifosfamide, carboplatin and etoposide [101]. None of these studies was specifically devoted to elderly patients, but each included patients older than 60 years. ORRs ranged from 31 to 86%. However, in the absence of subsequent consolidation treatment, the median PFS and OS after these treatments were disappointing and did not exceed 6 and 12 months, respectively.

Recent advances in DLBCL have been used to evaluate the activity of innovative agents in PCNSL, mainly in refractory/relapsing (R/R) tumors, with notably targeted therapies and immunotherapy, such as imids [102,103,104,105,106,107], ibrutinib [108,109,110,111] and anti-PD-1 [112,113,114]. As these novel agents demonstrate promising efficacy in term of objective response rate and good safety profiles in clinical trials or retrospective studies without limitation of age they are excellent candidates to be incorporated in 1st line induction and/or maintenance treatment in the near future and could benefit elderly patients.

### 4.4. The Oldest Patients

Patients aged 80 years or older represent up to 20% of all PCNSL patients [6]. However, data concerning this age population are very limited. Many clinicians are reluctant to treat the oldest patients, as illustrated in clinical trials without an upper limit of age showing that this patient group was underrepresented. A retrospective review of all PCNSL patients aged 80 years or older treated at MSKCC over almost 20 years, between 1993 and 2011 [19], reported 23 patients with a median age of 82 years (range from 80 to 90 years) and a median KPS of 50 (range from 30 to 90 years), mostly treated with HD-MTX-based polychemotherapy. The initial MTX dose was 3.5 g/m^2^ in 17 patients, while the other 5 patients started receiving doses ranging from 1 to 2.5 g/m^2^. Subsequent dose reduction was required in 14% (*n* = 3) of the patients because of elevations in creatinine level (*n* = 2) and one grade II pleural effusion; however, in 2 patients, this dose reduction was temporary. Six patients received cytarabine as consolidation treatment. The ORR was 62.5%, median PFS was 6.5 months, and median OS was 7.9 months. The 2 and 3-year OS were 33% and 17%, respectively. In a multivariate analysis, the only achievement of CR was identified as an independent age-survival predictor (adjusted HR 0.3, *p* = 0.04). Ten patients had grade III/IV toxicities (mainly myelosuppression), and one toxic death was reported.

Comparatively, the French LOC network database registered 120 consecutive newly diagnosed PCNSL patients older than 80 years between 2011 and 2016 [6]. A preliminary report based on 110 elderly patients from this database, with a median age of 83 years (range: 80–92), 1st line HD-MTX was administered in 77% (*n* = 85) at a median dose of 3 g/m^2^ (range from 0.5 to 5.0), while 12% were treated with other chemotherapy protocols and 11% were treated only with palliative care [115]. In the treated patients, the ORR was 46%, with median PFS and OS of 5 and 8 months, respectively. Patients treated with HD-MTX-based therapy had a significantly better outcome. Forty-six percent of grade III/IV toxicities were observed in patients treated with HD-MTX-based therapy, including 15% with infection, 13% with cytopenia, 11% with acute renal failure and 8% with elevated liver enzymes. Toxic death was reported in 13% of cases.

These reports show that HD-MTX is also a feasible and active treatment in the oldest patients but requires adapted doses according to urinary clearance and comorbidities. For patients unfit for HD-MTX treatment, due to impaired renal function or other comorbidities, temozolomide chemotherapy may be a treatment option. In a series of 17 elderly patients (6 in the oldest group) treated with temozolomide monotherapy as 1st line therapy, 29.4% had prolonged responses for at least 12 months and survived for more than 24 months with good tolerance [116].

WBRT alone can also be considered as a therapeutic option in the first-line for unfit patients and induces a high response rate. However, responses are most often of short duration [71] and given the high risk of neurotoxicity in elderly patients, we rather recommend chemotherapy options in this setting.

**Figure 1:**
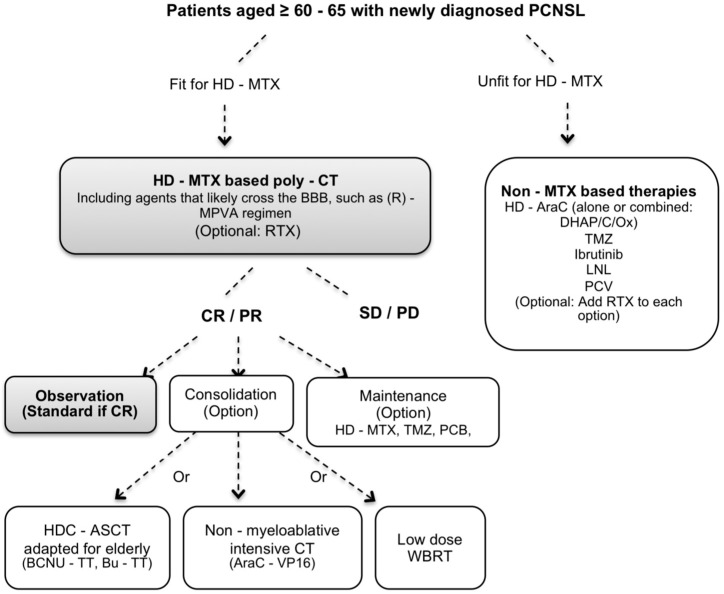
Working algorithm proposed by the authors of the present review for the 1st line treatment of elderly patients with PCNSL. Treatments considered standard are highlighted in gray. BBB: brain-blood barrier, CT: chemotherapy, CR: complete response, DHAP: dexamethasone + cytarabine + platinol, DHAC: dexamethasone + cytarabine + carboplatine, HD-AraC: high-dose cytarabine, HD-MTX: high-dose methotrexate, HDC-ASCT: high-dose chemotherapy conditioning with autologous stem cell transplantation, LNL: lenalidomide, MPVA: HD-MTX, procarbazine, vincristine and cytarabine, PCNSL: primary central nervous system lymphoma, PCV: procarbazine + CCNU + vincristine, PD: progressive disease, PR: partial response, RTX: rituximab, SD: stable disease, TMZ: Temozolomide, WBRT: Whole-brain radiotherapy.

**Figure 2:**
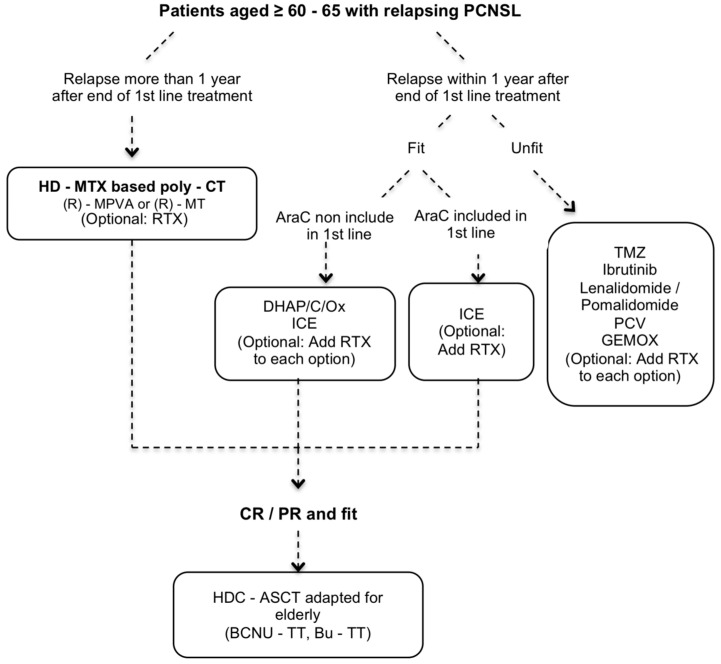
Working algorithm proposed by the authors of the present review for the management of elderly patients with relapsing PCNSL. BBB: brain blood barrier, CT: chemotherapy, CR: complete response, DHAP: dexamethasone + cytarabine + platinol, DHAC: dexamethasone + cytarabine + carboplatine, DHAOx: dexamethasone + cytarabine + oxaliplatine, GEMOX: gemcitabine + oxaliplatine, HD-MTX: high-dose methotrexate, HDC-ASCT: high-dose chemotherapy conditioning with autologous stem cell transplantation, ICE: ifosfamide + carboplatin + etoposide, LNL: lenalidomide, MPVA: HD-MTX + procarbazine + vincristine + cytarabine, MT: methotrexate + temozolomide, PCNSL: primary central nervous system lymphoma, PD: progressive disease, PR: partial response, RTX: rituximab, SD: stable disease, TMZ: Temozolomide.

Finally, we propose a working algorithm for the management of elderly patients with newly and relapsing PCNSL (Figure 1 and Figure 2, respectively). This algorithm represents authors’ preferred choices and not guidelines as we lack controlled data from prospective studies.

## 5. Conclusions

Elderly patients represent the majority of PCNSL patients with an increasing incidence over time. Although the overall prognosis is worse in elderly patients than in younger patients, the patients in the older age group should not be deprived of vigorous treatment because a high proportion of patients may achieve remission with clinical improvement, and prolonged survival may be expected in a substantial minority of patients. Due to a high risk of severe delayed neurotoxicity of combined radiochemotherapy treatment, HD MTX-based polychemotherapy alone as initial treatment is largely recommended.

Several factors contribute to the poor prognosis of the elderly: a high frequency of early deaths related to fragile functional status and comorbidities, a lower rate of CR compared to younger patients, and short-lasting remission and treatment-related toxicities. All these points represent important issues to consider for improving the management of elderly patients. Advances will come from promising novel agents and their combination with current approaches during different phases of treatment, to increase the response rate and outcome. Other perspectives will come from individualized treatments according to toxicity risk and prognosis through the use of geriatric indicators, comorbidity assessments, nutritional scales and predictive biomarkers. Hence, clinical trials, as well as translational research dedicated to this population, need to be strongly encouraged.

## Figures and Tables

**Table 1:** Main studies evaluating chemotherapy alone dedicated to newly diagnosed PCNSL in the elderly.

Author	Type	N	Median Age (Range)	Induction	Consolidation	Maintenance	CR % I/M or C ^1^	PFS OS mo	Toxic Deaths %
Hoang-Xuan 2003 [9]	Phase II	50	72 (60–81)	HD-MTX IV + IT, IT ARAC, CCNU, PCB	None	HD-MTX IV + IT, IT ARAC, CCNU, PCB	42	7 14.3	4
Omuro 2007 [53]	Retrospective	23	68 (60–79)	HD-MTX, TMZ	None	HD-MTX, TMZ	30/61	8 35	4
Zhu 2009 [54]	Retrospective	31	74 (70–85)	HD-MTX	None	HD-MTX	60	7.1 37	0
Illerhaus 2009 [55]	Phase II	30	70 (57–79)	HD-MTX, CCNU, PCB	None	None	44.4	5.9 15.4	6
Fritsch 2011 [56]	Phase II	28	75 (65–83)	HD-MTX, RTX, PCB, CCNU	None	None	64	16 18	7
Roth 2012 [8]	Retrospective	66	≥70 (NR)	HD-MTX based CT	None	None	64	13.9 26.7	9
					WBRT		75	24.1 29.3	
Olivier 2014 [57]	Phase I	35	65 (61–70)	MTX, VIND, IDA	None	None	17	13 19	8.5
Omuro 2015 [22]	Phase II randomized	48	73 (60–85)	HD-MTX, TMZ	None None	None	38	6 14	10
		47	72 (60–84)	HD-MTX, PCB, VCR, ARAC			53	9.5 31	6
Pulczynski 2015 [58] ^2^	Phase II	27	70 (66–75)	RTX, HD-MTX, TMZ, IFOS, IV + IT ARAC, VIND	None	TMZ	69/58	14 NA	15
Schorb 2017 [59]	Retrospective	15	70 (66–75)	HD-MTX based CT	HDC-ASCT (BCNU-TT, Bu-TT + Cy, TT)	None	27/73	NA NA	4
Fritsch 2017 [60]	Phase II	107	73 (66–85)	HD-MTX, RTX, PCB + CCNU	None	PCB	35.5	10.3 20.7	8
Houillier 2017 [11]	Retrospective	90	68 (60–87)	RTX, HD-MTX, PCB, VCR	3 cycles ARAC	None	55	10 28.1	6
Faivre 2019 [61]	Retrospective	10	67 (61–76)	MTX, PCB, VCR ± RTX	None	TMZ [6]	60/80	57 63	0
Vu 2019 [62]	Retrospective	13	77 (70–86)	MTX, RTX ± TMZ	None	LNL (NR)	85	29.4 31.6	0
Schorb 2020 [24]	Pilot trial	14	74 (69–79)	RTX, HD-MTX, ARAC	HDC-ASCT (Bu-TT)	None	29/85	NA NA	0

^1^ CR after induction (I) and maintenance (M) or consolidation therapy differenced, if reported. ^2^ Report only elderly patients results for studies made on all age population, ARAC: cytarabine, BCNU-TT: carmustine + thiotepa, Bu-TT: busulfan + thiotepa, CCNU: lomustine, Cy: cyclophosphamide, IFOS: Ifosfamide, HD: high dose; LNL: lenalidomide, MTX: methotrexate, PCB: procarbazine, RTX: rituximab, TMZ: temozolomide, VCR: vincristine, VIND: vindesin. IV intravenous, IT intrathecal. CR: complete response, OS: overall survival, PFS: progression-free survival, NA: not achieved, NR: not reported.

**Table 2:** Ongoing clinical trials for elderly patients with newly diagnosed PCNSL.

Clinical Trial for First-Line	Design	Phase	*n*	Age	Outcomes
Induction therapy					
NCT02836158	RTX, IDA, ARAC, MTX + IT RTX, MTX, ARAC	2	100	60–75	3-years OS
Induction (I) + Consolidation (C) treatment					
NCT03569995(CREMA)	I: RTX, MTX + C: RTX, ARAC	2	35	≥60	2-years PFS PFS, OS, FAE, TTF
NCT01399372	I: RTX, MTX, PCB, VCR + C: ARAC or Low dose WBRT	2	91	≥18	PFS OS, Response rate, Quality of life, Neurocognitive function
DRKS00011932	I: RTX, MTX, ARAC + C: HDT-ASCT (RTX, BU, TT)	2	51	≥65	1-year PFS CR rate, OS, PFS, Neurotoxicity, FAE
Induction (I) + Maintenance (M) treatment					
NCT03495960 (FIORELLA)	(A) I: RTX, MTX, PCB + M: PCB or LNL	2	208	≥70	2-years PFS TTF, Response Rates, OS, FAE, Relapse rates and patterns, Neurotoxicity
	(B) I: WBRT- RTX, TMZ + M: TMZ				
Maintenance treatment					
NCT02623010	Ibrutinib	2	30	60–85	PFS OS
NCT04627753 (LEMON-C)	RTX-LNL	2	30	≥65	1-year PFS 2-years PFS, OS, Overall response, Toxicity profiles
NCT04022980	Nivolumab (After HD-MTX based chemotherapy)	1	20	≥65	Dose-limiting toxicity, 2-years PFS PFS, OS, Response Rates, Conversion Rate (Partial to Complete Response)
NCT02313389 (BLOCAGE-1)	M: RTX, MTX, TMZ or Observation (After HD-MTX based polychemotherapy)	3	295	≥60	PFS OS, Toxicity, Cognitive functions, Quality of life

ARAC: cytarabine, CCNU: lomustine, CR: complete response, IDA: idarubicin, MTX: methotrexate; PCB: procarbazine, RTX: rituximab, TMZ: temozolomide, VCR: vincristine, VIND: vindesin. CR: complete response, OS: overall survival. PFS: progression-free survival. FAE: frequency of adverse effects. TTF: time to failure. IELSG: International Extranodal Lymphoma. Study Group.
